# Evaluating knowledge, attitude, and physical activity levels related to cardiovascular disease in Egyptian adults with and without cardiovascular disease: a community-based cross-sectional study

**DOI:** 10.1186/s12889-024-18553-3

**Published:** 2024-04-22

**Authors:** Alaa Ramadan, Heba Aboeldahab, Mohamed Nabih Bashir, Mohamed Mohamed Belal, Ahmed Wageeh, Ahmed Atia, Mohamed Elbanna, Tala Jouma Alhejazi, Mohamed Abouzid, Hady Atef, Esraa Khalid, Osama Ahmed Abd Elaziz, Mariam Ibrahim Eldeeb, Doha Omar Kamel Omar, Neveen Refaey, Amr Setouhi, Mohammed AK

**Affiliations:** 1 Faculty of Medicine, South Valley University, Qena, Egypt; 2 Biomedical Informatics and Medical Statistics Department, Medical Research Institute, Alexandria University, Alexandria, Egypt; 3 Faculty of Medicine, Alexandria University, Alexandria, Egypt; 4 Faculty of Medicine, Menoufia University, Menoufia, Egypt; 5 Faculty of Medicine, Cairo University, Cairo, Egypt; 6 Cornwall Partnership NHS Foundation Trust, Bodmin, UK; 7 School of Allied Health Professions, Keele University, Staffordshire, UK; 8 Faculty of Physical Therapy, Cairo University, Cairo, Egypt; 9 Faculty of Medicine, Al-Azhar University, Cairo, Egypt; 10 Faculty of Medicine, University of Aleppo, Aleppo, Syrian Arab Republic; 11 Faculty of Medicine, Misr University of Sciences and Technology, Cairo, Egypt; 12 Faculty of Medicine, Suez Canal University, Ismailia, Egypt; 13 Department of Physical Pharmacy and Pharmacokinetics, Faculty of Pharmacy, Poznan University of Medical Sciences, Poznan, Poland; 14 Doctoral School, Poznan University of Medical Sciences, Poznan, Poland; 15 Department of Women’s Health, Faculty of Physical Therapy, Cairo University, Cairo, Egypt; 16 Cardiovascular Medicine, Minia University, Minya, Egypt; 17 Internal Medicine, Faculty of Medicine, Qena University Hospital, Qena, Egypt

**Keywords:** Awareness, Health promotion, KAP, Cardiovascular disease, Egypt

## Abstract

**Background:**

Cardiovascular disease (CVD) represents a significant health challenge in Egypt, yet there exists limited understanding regarding the knowledge, attitudes, and physical activity levels associated with CVD. These factors play a pivotal role in developing effective prevention and management strategies. Hence, this cross-sectional study aimed to evaluate Egyptian adults’ knowledge, attitudes, and physical activity (KAP) levels.

**Methods:**

Data were collected using a previously validated questionnaire encompassing demographic characteristics, CVD knowledge (including risk factors and symptoms), attitudes toward CVD, and self-reported physical activity levels. The survey was distributed among social media channels, and trained researchers administered the questionnaire via face-to-face interviews with adult patients with and without CVD admitted to Cairo University Hospital clinics.

**Results:**

The study involved 591 participants, of whom 21.7% had CVD. Overall, participants exhibited poor knowledge regarding CVD, with a mean score of 21 ± 7 out of 40, equivalent to 52.5%. Attitudes toward CVD were moderate, with a mean score of 66.38 ± 8.7 out of 85, approximately 78%. Physical activity levels per week were also moderate, averaging 1188 MET-min with a range of 1121–18,761. Subgroup analysis revealed that individuals with CVD had lower average knowledge, attitude, and physical activity levels than those without CVD. Working in the healthcare field was a predictor of higher knowledge score (standard error (SE) 5.89, 95% confidence interval (CI) 4.61 to 7.17, *P* < 0.001), while those with CVD and smokers were predictors of lower attitude score (SE -4.08, 95% CI -6.43 to -1.73, *P* < 0.001) and (SE -2.54, 95% CI -4.69 to -0.40, *P* = 0.02), respectively.

**Conclusion:**

The study findings highlight a significant disparity in knowledge, attitudes, and physical activity levels related to CVD in Egypt. Targeted interventions aimed at improving awareness, fostering positive attitudes, and promoting physical activity among individuals at risk for CVD are crucial for effective prevention and management.

**Supplementary Information:**

The online version contains supplementary material available at 10.1186/s12889-024-18553-3.

## Introduction

Cardiovascular disease (CVD) is a major source of morbidity and mortality around the world [1]. It includes coronary artery disease, stroke, and heart failure, all of which can have severe consequences for individuals, families, and healthcare systems [2]. According to estimates, 17.9 million deaths worldwide in 2016 were attributed to CVDs, or 31% of all deaths. The World Health Organization states that heart attacks and strokes account for 85% of these deaths. More than 75% of deaths from CVDs occur in low- and middle-income nations [3, 4].

Several individual and socio-environmental factors may be contributing to the rising CVD epidemic: a sedentary lifestyle, high cholesterol, diabetes, obesity, smoking, high-fat diets, and excessive alcohol use are among the modifiable risk factors for CVD [5, 6]. According to the Global Burdens of Disease Report, high blood pressure and smoking were found to be the leading global risk factors for early death and disability across all age groups [1, 7]. As a result, the morbidity and mortality of the disease can be avoided by creating more targeted population-based prevention programs. Since it has been shown that awareness of CVD and its risk factors is a precondition for changing people’s health behaviors and lifestyles, having a solid understanding of these factors can help people reduce their risk. Furthermore, knowing the warning signals of a heart attack or stroke might help patients arrive at the hospital early and have better results [8, 9].

Numerous global studies demonstrate the low level of population-level knowledge, attitude, and physical activity (KAP) on CVDs and emphasize the significance of increasing the KAP level to lessen the burden of CVDs [10–12]. Studies on Egyptians’ awareness of heart disease and its modifiable risk factors are scarce. Therefore, in this study, we aimed to evaluate the knowledge, attitudes, and physical activity related to cardiovascular disease among Egyptian adults, both with and without cardiovascular disease. By conducting a community-based cross-sectional study, we seek to understand the prevalence and patterns of these factors in the general population. The findings of this study could provide valuable insights for healthcare professionals and policymakers in developing targeted interventions and strategies to promote cardiovascular health in Egypt.

## Methods

### Ethical considerations

Ethical approval was obtained from the Ethical Research Committee of Cairo University, Egypt (012/004122). Informed consent was obtained from all participants before their inclusion in the study, ensuring confidentiality and privacy of their information.

### Study design and eligibility criteria

We conducted a cross-sectional comparative study adhering to the STROBE guidelines [13] from September 11 to October 16, 2023. The inclusion criteria included adults who could understand Arabic residents of Egypt. Participants were with and without CVD (hence after, referred to as CVD and non-CVD for simplicity) and had to be ≥ 18 years old. The exclusion criteria that the inclusion criteria cannot meet are those with a physical disability. The exclusion criteria included all residents under 18 years old and those who refused to participate in the study or were uncollaborative during the interview.

### Sample size determination

The total sample size required for this study was calculated based on sample size calculation methods for a single proportion using an online sample size calculator (Open Epi) [14]. Assuming a CVD prevalence of 36% [15], a minimum sample size of 120 participants was required to detect a similar proportion rate with 95% confidence intervals and 5% precision. This sample size was determined to adequately capture the variability within each group and ensure statistical significance in the study outcomes. Moreover, the design effect accounted for the stratified sampling methodology employed, which enhanced the representativeness and generalizability of the findings across different demographic segments.

### Data collection

Two main strategies were employed to collect data for this study. First, we developed an online link to a web-based questionnaire using “Google Forms.” A Plain Language Information Statement (PLIS) and Consent Form were provided on the initial screen. The PLIS included information about local country-specific investigators who facilitated responses to relevant questions during data collection. After providing consent and meeting age and other inclusion criteria, participants could proceed to subsequent pages to answer the questionnaire.

The second approach involved direct interviews with individuals from clinics associated with Cairo University Hospital. Ethical approval was obtained, allowing for seamless collaboration and efficient data collection. In both strategies, participant selection was entirely random, ensuring the inclusion of diverse perspectives from both patient groups. This approach enhances the overall robustness and reliability of the study findings. Notably, this approach was also used in previous studies [12, 16].

### The questionnaire

The validated survey instrument initially originated from a previous study conducted in Lebanon by Machaalani et al. [12] and was adapted to suit the Egyptian population for this research endeavor. Rigorous review processes were undertaken to ensure the validity and reliability of the KAP scores. Furthermore, meticulous attention was given to defining all variables within the questionnaire, including CVD-related physical activity, smoking status, social status, and obesity, thereby facilitating precise data collection and interpretation. Physical activity levels were calculated based on responses provided in the IPAQ Sect. [17], allowing for stratification and analysis of participants’ activity levels.

The questionnaire was divided into the following sections:

i. Sociodemographic and other patient-related characteristics: 11 questions assessing the patient’s sex, age, marital status, occupation, educational level, social and economic status, working in the healthcare field, suffering from cardiovascular diseases (diagnosed by a specialist), smoking status, Medical history of chronic disease and Source of information.
ii. CVD knowledge: 40 questions assessing patients’ knowledge of CVD and its consequences, symptoms of coronary heart disease (CHD), CVD risk factors, CVD risk levels (desirable values of high-density lipoprotein cholesterol (HDL-c), low-density lipoprotein cholesterol (LDL-c), fasting glycemia, normal blood pressure (BP) ranges, and normal BMI).
iii. CVD Attitude: 17 questions covered regular lipid profile, glycemia, and blood pressure measurements; diet plan; salt intake; treatment adherence; maintenance of normal body weight; and exercise. After that, that was reviewed by two experts in the CVD field with experience of > 10 years. The survey on CVD-related attitudes assessed participants’ perspectives through 17 items, each rated on a Likert scale ranging from 1 (strongly disagree) to 5 (strongly agree).
iv. CVD Physical Activity: these seven questions include walking, sitting, and intense and mild physical activity during the last three months. It inquires explicitly about the frequency and length of walking, the amount of time spent sitting during the workday, and the frequency and duration of both strenuous and moderate physical activities. International Physical Activity Questionnaire (Short Form) IPAQ is a validated instrument designed primarily for population surveillance of adults. It has been developed and tested for use in adults (age range of 15–69 years) [17].

Regarding the scoring, the knowledge and attitude sections were scored so that every correct answer was granted 1 point and each wrong answer a 0. A 5-point Likert scale was adopted in which ‘strongly disagree’ was given 1 point and ‘strongly agree’ was given 5 points for all items. The overall KAP score was calculated from the sum of the points granted, where the cut-off value was the median for each section based on previously reported KAP investigations [18, 19].

### Statistical analysis

The Collected data were analyzed using RStudio software. The cumulative replies to each question were reported along with their respective percentages. Data were represented as frequencies and proportions for the nominal variables. Normally distributed continuous variables were reported as mean (± SD) or median (interquartile range, IQR) for not normally distributed data. Scores of KAP were computed. Items included for the knowledge score were 40, 17 for the attitude score, and 7 for the physical activity score. The tests used were the Mann–Whitney U and Kruskal-Wallis tests, which were tests with ANOVA. In addition, the correlation between the three ABK scores was tested using the Pearson or Spearman correlation test. A multivariate analysis was conducted to test factors affecting each of the three scores in the population, and its results were reported as standard error (SE) and their 95% confidence intervals (CI). The significance level was set at 5%.

## Results

### Demographic characteristics

A total of 591 participants were included in this study. Of them, 21.7% had a CVD. A statistically significant higher percentage of males was found in the CVD group (76.6%) compared to the non-CVD group (44.9%)(*P* < 0.001). Age significantly differed between the two groups (*P* < 0.001), with the CVD group having a higher median age (25 years, range 19–72) compared to the non-CVD group (22 years, range 16–65). Age categories also exhibited a significant difference (*p* < 0.001), with a higher proportion of young individuals (< 45 years) in the non-CVD group (97.2%) compared to the CVD group (78.9%). In terms of marital status, a total of 59.7% of the participants reported being single. A higher percentage of the CVD group reported being married compared to the non-CVD group (88.3% vs. 25.3%, respectively) (*P* < 0.001).

The total prevalence of current smokers was 14.7%. The CVD group (36.7%) showed a significantly higher proportion of current smokers compared to non-CVD (8.6%) (*P* < 0.001). Occupation, educational level, social and economic status, and healthcare field employment were all significantly associated with CVD with a P-value < 0.001. A higher percentage of CVD patients reported being employed (82.8%), having a university educational level (81.3%), lower social and economic status (87.5%), and not working in the healthcare field (96.9%). Several medical history variables showed a significantly higher prevalence of these conditions among individuals with CVD compared to non-CVD individuals, including diabetes mellitus (32.8% vs. 2.4%), hypertension (60.9% vs. 5.2%), high cholesterol (22.7% vs. 4.1%), previous surgeries (22.7% vs. 14.5%), lung disease (16.4% vs. 3.5%), liver disease (9.4% vs. 0.9%), and renal disease (7% vs. 1.5%).

In this section, sources of information about cardiovascular diseases, attending medical conferences, healthcare worker status, and specialized health journals were significantly associated with CVD status with a P-value < 0.001, with a lower proportion of CVD patients reporting these sources of information. Other demographic details are in Table 1.

**Table 1:** Bivariate analysis of the included respondents’ demographics and other features (CVD patients vs. non-CVD participants; *N* = 591)

Variable	Non-CVD (*N* = 463)	CVD (*N* = 128)	Total	P-value
Age, Median (IQR)	22(21–25)	25(19–72)	23(16–72)	< 0.001
*Age groups, n (%)*				
Young (< 45)	450(97.2)	101(78.9)	551(93)	< 0.001
Adult (45–65)	11(2.4)	23(18)	34(5.8)	
Elderly (> 65)	2(0.4)	4(3.1)	6(1)	
*Sex, n (%)*				
Male	208(44.9)	98(76.6)	306(51.8)	< 0.001
Female	255(55.1)	30(23.4)	285(48.5)	
*Marital Status, n (%)*				
Single	343(74.1)	10(7.8)	353(59.7)	< 0.001
Married	117(25.3)	113(88.3)	230(38.9)	
Divorced	3(0.6)	1(0.8)	4(0.7)	
Widow	0	4(3.1)	4(0.7)	
*Occupation, n (%)*				
Employed	218(47.1)	106(82.8)	324(54.8)	< 0.001
*Educational level, n (%)*				
No Formal Education	0	9(7)	9(1.5)	< 0.001
Primary Level	0	3(2.3)	3(0.5)	
Preparatory Level	0	5(3.9)	5(0.8)	
Secondary Level	40(8.6)	6(4.7)	46(7.8)	
University Level	389(84)	104(81.3)	493(83.4)	
Post-graduate	34(7.3)	1(0.8)	35(5.9)	
*Socioeconomic status, n (%)*				
Low	67(14.5)	112(87.5)	179(30.3)	< 0.001
Moderate	372(80.3)	15(11.7)	387(65.5)	
High	24(5.2)	1(0.8)	25(4.2)	
*Are you working in the healthcare field?, n (%)*				
Yes	184(39.7)	4(3.1)	188(31.8)	< 0.001
*Are you currently a smoker?, n (%)*				
Yes	40(8.6)	47(36.7)	87(14.7)	< 0.001
*Medical history, n (%)*				
Diabetes Mellitus	11(2.4)	42(32.8)	53(9)	< 0.001
Hypertension	24(5.2)	78(60.9)	102(17.3)	< 0.001
High Cholesterol	19(4.1)	29(22.7)	48(8.1)	< 0.001
Previous Surgeries	67(14.5)	29(22.7)	96(16.2)	0.026
Obesity	54(11.7)	23(18)	77(13)	0.061
Lung Disease	16(3.5)	21(16.4)	37(6.3)	< 0.001
Thyroid Disease	8(1.7)	6(4.7)	14(2.4)	0.51
Liver Disease	4(0.9)	12(9.4)	16(2.7)	< 0.001
Renal Disease	7(1.5)	9(7)	16(2.7)	< 0.001
*Source of Information, n (%)*				
TV and Radio	119(25.7)	29(22.7)	148(25)	0.481
Attending Medical Conferences	78(16.8)	1(0.8)	79(13.4)	< 0.001
Healthcare Worker	219(47.3)	111(86.7)	330(55.8)	< 0.001
Specialized health Journals	146(31.5)	10(7.8)	156(26.4)	< 0.001
Experience of Cardiac Patient	119(25.7)	29(22.7)	148(25)	0.481
Newspapers and General Magazines	82(17.7)	15(11.7)	97(16.4)	0.105

All statistical tests here were conducted using the Chi-squared Test

### CVD-related KAP

The findings indicated that the overall CVD knowledge among all participants was poor grade, with a mean score of 21 ± 7 out of 40, equivalent to 52.5%. Participants demonstrated a moderate attitude concerning CVD, scoring a mean of 66.38 ± 8.7 out of 85, approximately 78%. Physical activity per week was also moderate according to IPAQ criteria [17], with a median of 1188 MET-min with a range of [0-19278]. When we examined each subgroup separately, we found that the average knowledge score related to CVD in CVD patients was lower compared to non-CVD responders. Specifically, it was 20 ± 6.9 out of 40 (representing a poor level), whereas non-CVD responders scored 21.37 ± 7 out of 40 (indicating a poor level as well), with the difference being statistically significant (*p* < 0.007). Likewise, the mean attitude score regarding CVD in CVD patients was significantly lower than in non-CVD responders. CVD patients scored an average of 62.8 ± 7.4 out of 85 (indicating a moderate level), while non-CVD responders scored 67.35 ± 8.7 out of 85 (also indicating a moderate level), with the difference being statistically significant (*p* < 0.001). Additionally, the average MET-min per week in CVD patients was significantly lower compared to non-CVD responders, standing at 1557 ± 2401 (indicating a moderate level) versus 2789 ± 3302 (also indicating a moderate level) respectively, and the difference was statistically significant (*p* < 0.001). For more details, refer to Tables 2 and 3.

**Table 2:** Bivariate analysis of participants’ distribution (N) (CVD vs. Non-CVD) based on subcategories (Poor, Moderate, Good) and KAP scores regarding CVD

Score	Grade	*Study Group*		*Total, n (%)*	*P-value*
		NON-CVD, n (%)	CVD, n (%)		
Knowledge	Fail	7(1.5)	7(5.5)	14(2.4)	< 0.001$
	Pass	210(45.4)	34(26.5)	244(41.3)	
	Outstanding	246(53)	87(68)	333(56.3)	
Attitude	Good	264(57.1)	34(26.5)	298(50.4)	< 0.001$
	Moderate	172(37.1)	87(68)	259(43.8)	
	Poor	27(5.8)	7(5.5)	34(5.8)	
Physical Activity	Low	130(28.6)	67(53.2)	197(34)	< 0.001$
	Moderate	324(71.4)	59(46.8)	383(66)	

$ Chi-square test was used to test KAP differences between individuals with and without CVD

**Table 3:** The mean range of the assessment scores

Variable	Value	Non-CVD	CVD	Total	P-value
Knowledge	*Mean (SD)*	21.37(7)	20(6.9)	21(7)	0.007*
Attitude	*Mean (SD)*	67.35(8.7)	62.8(7.4)	66.38(8.7)	< 0.001*
Physical Activity	*Median (Range)*	1371 [1163–17,230]	306 [207–6521]	1188 [1121–18,761]	< 0.001*

*Mann-Whitney test was used

### CVD-related knowledge

Among the population, 88.5% correctly acknowledged the relationship between CVD and the heart, while 84.4% understood that hypertension is a CVD risk factor. However, 57.7% believe that most cancer is not a CVD risk factor, and 69.4% incorrectly think that CVD is not the primary cause of death in diabetic patients. For detailed results, please refer to Table S1.

### CVD-related attitude

A significant proportion of participants expressed a strong willingness to exercise (4.3 ± 1.0) and change eating habits easily (3.9 ± 1.0). The majority agreed that maintaining an average weight (2.7 ± 1.36), not smoking or being exposed to passive smoke (4.5 ± 1.1), and taking treatment as recommended by a doctor (4.3 ± 1.0) were essential for cardiovascular health. Participants showed hesitation in taking Hormone Replacement Therapy (HRT) (2.6 ± 1.5) and expressing resistance to traditional medicine preferences (3.2 ± 1.4). For more details, please refer to Table S2.

### CVD-related physical activity

A larger number of the CVD group exhibited moderate physical activity in specific categories, including those who were either young (< 45 years) or older (> 65 years), single, had no formal education, were post-graduates, and had a moderate socioeconomic status. Conversely, non-CVD participants showed more individuals with low physical activity levels in all remaining categories.

### Correlation among KAP score parameters

Among CVD patients, there were positive but nonsignificant correlations between Attitude and Knowledge (*r* = 0.104, *P* = 0.242), Attitude and Physical Activity (*r* = 0.08, *p* = 0.387), and Knowledge and Physical Activity (*r* = 0.101, *P* = 0.275). Non-CVD patients showed a significant positive correlation between Attitude and Knowledge (*r* = 0.12, *P* = 0.01), but the correlations between Attitude and Physical Activity and Knowledge and Physical Activity were not significant. These findings highlight distinct patterns of association between KAP parameters in individuals with and without cardiovascular disease Table 4.

**Table 4:** Correlation among KAP score parameters

Score	Subgroups	Spearman Correlation		Pearson Correlation	
		r	P-value	r	P-value
Attitude Versus Knowledge	Total	0.145	< 0.001	0.131	0.001
	No - CVD	0.101	0.029	0.12	0.01
	CVD	0.166	0.062	0.104	0.242
Attitude Versus Physical Activity	Total	0.047	0.254	0.045	0.281
	No - CVD	-0.006	0.904	0.25	0.588
	CVD	0.08	0.387	0.08	0.387
Knowledge Versus Physical Activity	Total	0.063	0.127	0.063	0.13
	No - CVD	0.024	0.601	0.047	0.31
	CVD	0.202	0.029	0.101	0.275

### Sociodemographic characteristics of CVD patients and non-CVD participants with poor knowledge, poor attitude, and low physical activity score

CVD individuals had a higher occurrence of poor CVD knowledge scores compared to non-CVD patients across various sociodemographic characteristics. However, there are some exceptions to this trend: participants aged over 65 (50% vs. 100%), divorced participants (0% vs. 66.7%), those with a secondary educational level (50% vs. 62.5%), and individuals with high socioeconomic status (0% vs. 29.2%). Among the characteristics examined, a greater number of CVD participants displayed poor attitudes towards CVD in the following groups: females, adults aged 45–65, those who were single or married, individuals with no formal education or post-graduate qualifications, employed individuals, those with a moderate socioeconomic status, and non-smokers. In contrast, non-CVD participants exhibited a higher number of individuals with poor attitudes towards CVD in all other categories. Table 5 represents the CVD and non-CVD patients with poor knowledge scores, poor attitudes, and low physical activity categorized by their sociodemographic characteristics.

**Table 5:** According to sociodemographic status, the percentage (%) of Egyptian CVD patients and non-CVD subjects (Control) with poor knowledge, poor attitudes, and low physical activity about CVD

Variable			% Poor Knowledge	% Poor Attitude	% Low Physical activity
Gender	Male	Non-CVD	51.4	5.8	46
		CVD	67.3	5.1	30.8
	Female	Non-CVD	54.5	5.9	48.6
		CVD	70	6.7	23.3
Age	< 45	Non-CVD	52.9	6	28.4
		CVD	69.3	5.9	57.3
	45–65	Non-CVD	54.5	0	41.7
		CVD	65.2	4.3	23.1
	> 65	Non-CVD	100	0	0
		CVD	50	0	33.3
Marital Status	Single	Non-CVD	51.6	6.7	45.2
		CVD	80	10	60
	Married	Non-CVD	57.3	3.4	26.5
		CVD	67.3	5.3	2.7
	Divorced	Non-CVD	66.7	0	66.7
		CVD	0	0	0
	Widowed	Non-CVD	0	0	0
		CVD	75	0	0
Education	No Formal Education	Non-CVD	0	0	0
		CVD	66.7	11.1	0
	Primary Level	Non-CVD	0	0	0
		CVD	66.7	0	0
	Preparatory Level	Non-CVD	0	0	0
		CVD	60	0	0
	Secondary Level	Non-CVD	62.5	10	52.5
		CVD	50	0	0
	University Level	Non-CVD	53.2	5.7	39.1
		CVD	69.2	4.8	7.7
	Post-graduate	Non-CVD	42.2	2.9	44.1
		CVD	100	100	100
Occupation	Employed	Non-CVD	49.1	5.5	35.3
		CVD	67.9	6.6	6.6
	Unemployed	Non-CVD	56.7	6.1	45.3
		CVD	68.2	0	9.1
Social and Economic Status	Low	Non-CVD	61.2	7.5	11.9
		CVD	68.8	5.4	0
	Moderate	Non-CVD	53.2	5.6	45.7
		CVD	66.7	6.7	60
	High	Non-CVD	29.2	4.2	41.7
		CVD	0	0	0
Smoking Status	Smoker	Non-CVD	52.2	5.9	41.4
		CVD	67.9	4.9	8.6
	Non-smoker	Non-CVD	62.5	5	32.5
		CVD	68.1	6.4	4.3

### Factors affecting knowledge and attitude scores of the enrolled CVD patients and non-CVD participants

We used the bivariate analysis to identify the factors influencing CVD and non-CVD participants’ knowledge and attitude scores. The knowledge score showed no significant association with any factors examined in the CVD or non-CVD groups Table S3. In non-CVD participants, the attitude score exhibited a significant association with smoking status (P-value = 0.031). No other characteristics were associated with the attitude score in the non-CVD or CVD groups Tables S3 and S4.

### Predictors of KAP among enrolled populations

**Fig. 1:**
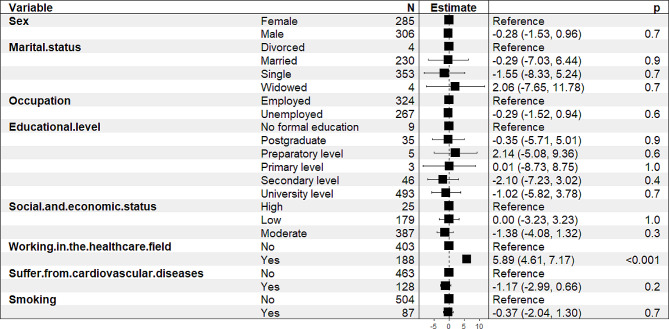
Multiple linear regression of knowledge score

Multiple linear regression was applied to identify predictors of knowledge score, which was significantly (*p* < 0.05) and positively associated with working in the health care field but negatively associated with sex, marital status, occupation, educational level, socioeconomic status, suffering from CVD and smoking (Fig. 1).

**Fig. 2:**
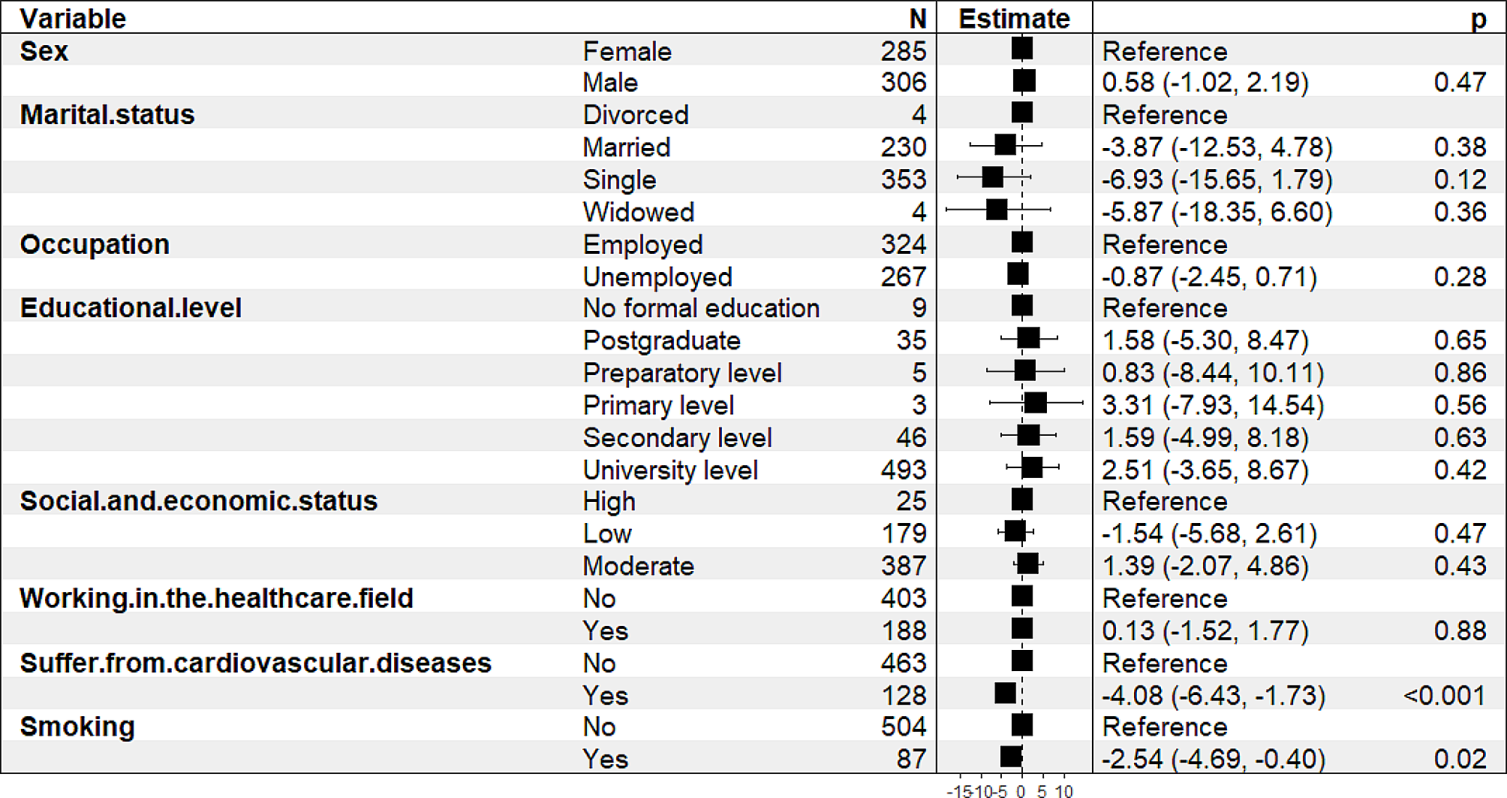
Multiple linear regression of attitude score

Attitude towards CVD was positively associated with suffering from CVD but negatively associated with working in the health care field, sex, marital status, occupation, educational level, socioeconomic status, and smoking (Fig. 2).

## Discussion

A total of 591 participants were included in the study. Our results showed that males, older age, marriage, smoking, diabetes, hypertension, and high cholesterol participants were significantly associated with CVD. Overall knowledge about CVD was poor, with a mean score of 52.5%. Attitudes toward CVD were moderate, with a mean score of 78%. Physical activity levels were also moderate on average. CVD patients had significantly lower knowledge, attitude, and physical activity scores than those without CVD. CVD patients were more likely to have poor knowledge across various demographics. Non-CVD participants had poorer attitudes in most categories except females, middle-aged, married, less educated, employed, moderate socioeconomic status, and non-smokers. Smoking was the only factor significantly associated with attitudes in non-CVD participants.

According to historical perspectives on CVD risk indicators, most risk factors were determined to be more prevalent in CVD patients than in non-CVD responders [20, 21]. Biological risk factors such as hypertension, dyslipidemia, diabetes mellitus, and obesity have been consistently identified as significantly more prevalent among individuals with CVD compared to those without the disease. Elevated blood pressure, abnormal lipid profiles, impaired glucose metabolism, and increased body mass index are established markers contributing to CVD’s pathogenesis and progression [22, 23]. Our findings indicated that the overall CVD knowledge among all participants was poor. In terms of attitudes, participants demonstrated a moderate level of attitude concerning CVD. Regarding physical activity per week, it was also at a moderate level. When we examined each subgroup separately, we found that the average knowledge score related to CVD in CVD patients was lower than non-CVD responders, unlike the Machaalani et al. study in which CVD patients showed more knowledge about CVD than non-CVD responders [12]. However, as was also noticed in other studies, the degree of knowledge in both sub-populations remained low. For instance, Wang et al. discovered that before obtaining health education and rehabilitation education, the knowledge levels of both the CVD and non-CVD groups were lower in the Chinese populations, and this is in line with the prevalence of CVD in China, which accounted for 46.74 of all deaths [24, 25]. According to Rosediani et al. Only a tiny percentage of women in Northeast Coast Malaysia were aware of unusual CVD symptoms such as nausea, jaw discomfort, and left shoulder pain [23]. Another study from Malaysia supports the premise of potentially lower CVD risk awareness. Ang et al. found that the incidence of sudden cardiac death was lower in East Malaysia compared to Western nations despite similar CVD prevalence. They attributed this to under-reporting and decreased healthcare accessibility in rural areas of East Malaysia [26]. In contrast, Nursyafiza et al. and Koohi et al., carried out in Kuantan and Tehran, respectively, revealed that participants had enough awareness of CVDs and their risk factors, which is questionable as the CVD-related death in Tehran is still relatively high according to the latest evidence [10, 11, 27]. Among the whole population in our study, 88.5% correctly acknowledged the relationship between CVD and the heart, while 84.4% understood that hypertension is a CVD risk factor. Likewise, other studies demonstrated that patients were mostly aware of smoking, diabetes, and hypertension as risk factors for CVD [10, 12]. However, in our study, 57.7% believe that most cancer is not a CVD risk factor, and 69.4% incorrectly think that cardiovascular disease is not the primary cause of death in diabetic patients. Doctors usually tend to concentrate on screening for menopausal disorders and reproductive malignancies, based on the assumption that women are less likely than males to get CVD [28]. This makes it more difficult for them to identify risk factors, create initiation therapy, diagnose heart problems like myocardial infarction, consult specialists, and give emergency care [29]. According to the Framingham Study, silent myocardial infarction accounts for a higher proportion of unexpected deaths in women who have never experienced a heart attack [30, 31]. Likewise, the mean attitude score regarding CVD in CVD patients was significantly lower than in non-CVD responders. However, attitudes in both groups stayed within the moderate level. Unlike our study, Wang et al. found that the CVD group had a significantly higher positive attitude than the non-CVD individuals [32]. Regarding physical activity, a larger number of the CVD group exhibited low physical activity in specific categories, including those who were either young (< 45 years) or older (> 65 years), single, had no formal education, post-graduates, and had a moderate socioeconomic status. Conversely, non-CVD participants showed more individuals with low physical activity levels in all remaining categories. According to numerous epidemiological research, higher levels of physical exercise are associated with a lower risk of developing cardiovascular disease (CVD). Frequent engagement in aerobic exercise, such as running, cycling, swimming, or fast walking, has been demonstrated to reduce the risk of cardiovascular disease (CVD)-related illnesses, including stroke. Improvements in blood pressure, lipid profiles, insulin sensitivity, vascular function, and general cardiovascular fitness are among the factors underpinning this protective impact [33–37].

## Strength and limitations

Both individuals with and without CVD were included in this study, allowing for comparisons between the groups. We used a validated questionnaire to comprehensively assess CVD-related knowledge, attitudes, and physical activity. Additionally, the questionnaire was administered through face-to-face interviews conducted by trained researchers with CVD patients. However, there are some limitations: the cross-sectional design prevents the inference of causality in relationships between variables; self-reported data on physical activity and medical history may be affected by recall or reporting biases; the study was conducted at a single center in one region of Egypt, which limits generalizability to other populations; the extensive number of questions in the survey may have burdened participants, potentially affecting the quality of responses due to fatigue or time constraints. Therefore, only 3% of the responses were collected online. The data collection strategy was extended to include on-site face-to-face interviews to address this. However, the availability of places for face-to-face interviews was mainly limited to the clinics of Cairo University Hospital, where ethical approval was obtained. This posed challenges for the research team in accessing different healthcare facilities. Researchers and policymakers should consider these limitations when interpreting the results and designing future studies.

## Research recommendation

Based on the study’s findings, several research recommendations can be made. Firstly, there is a need for targeted interventions to improve knowledge about CVD among both CVD patients and the general population. Additionally, efforts should be made to address misconceptions, such as the incorrect belief that cancer is not a CVD risk factor. Secondly, interventions should aim to improve attitudes towards CVD, particularly among CVD patients. Future research endeavors could expand the study’s geographical coverage to include multiple regions across Egypt, further enhancing the findings’ generalizability to the broader Egyptian population. Lastly, strategies to promote physical activity should be implemented, targeting populations with low physical activity levels, such as young and older individuals, those with lower education levels, and those with moderate socioeconomic status.

## Conclusion

All participants’ overall knowledge about CVD was poor, with an average score of 52.5%. Attitudes toward CVD were moderate, with a mean score of 78%. Physical activity levels were also moderate on average. CVD patients had lower knowledge, attitude, and physical activity scores than those without CVD. Risk factors like hypertension, dyslipidemia, diabetes, and obesity were more prevalent in CVD patients. The study highlighted the need for improved knowledge, attitudes, and physical activity levels to prevent and manage CVD.

### Electronic supplementary material

Below is the link to the electronic supplementary material.

Supplementary Material 1

## Data Availability

The data and materials used in this cross-sectional study are available upon request For the data contact the first author: dr. Alaa Ramadan Email: Alaaramadan251@gmail.com.
