# Publisher Correction: Evaluating knowledge, attitude, and physical activity levels related to cardiovascular disease in Egyptian adults with and without cardiovascular disease: a community-based cross-sectional study

**DOI:** 10.1186/s12889-024-18722-4

**Published:** 2024-05-02

**Authors:** Alaa Ramadan, Heba Aboeldahab, Mohamed Nabih Bashir, Mohamed Mohamed Belal, Ahmed Wageeh, Ahmed Atia, Mohamed Elbanna, Tala Jouma Alhejazi, Mohamed Abouzid, Hady Atef, Esraa Khalid, Osama Ahmed Abd Elaziz, Mariam Ibrahim Eldeeb, Doha Omar Kamel Omar, Neveen Refaey, Amr Setouhi, Mohammed AK

**Affiliations:** 1 Faculty of Medicine, South Valley University, Qena, Egypt; 2 Biomedical Informatics and Medical Statistics Department, Medical Research Institute, Alexandria University, Alexandria, Egypt; 3 Faculty of Medicine, Alexandria University, Alexandria, Egypt; 4 Faculty of Medicine, Menoufia University, Menoufia, Egypt; 5 Faculty of Medicine, Cairo University, Cairo, Egypt; 6 Cornwall Partnership NHS Foundation Trust, Bodmin, UK; 7 School of Allied Health Professions, Keele University, Staffordshire, UK; 8 Faculty of Physical Therapy, Cairo University, Cairo, Egypt; 9 Faculty of Medicine, Al-Azhar University, Cairo, Egypt; 10 Faculty of Medicine, University of Aleppo, Aleppo, Syrian Arab Republic; 11 Faculty of Medicine, Misr University of Sciences and Technology, Cairo, Egypt; 12 Faculty of Medicine, Suez Canal University, Ismailia, Egypt; 13 Department of Physical Pharmacy and Pharmacokinetics, Faculty of Pharmacy, Poznan University of Medical Sciences, Poznan, Poland; 14 Doctoral School, Poznan University of Medical Sciences, Poznan, Poland; 15 Department of Women’s Health, Faculty of Physical Therapy, Cairo University, Cairo, Egypt; 16 Cardiovascular Medicine, Minia University, Minya, Egypt; 17 Internal Medicine, Faculty of Medicine, Qena University Hospital, Qena, Egypt

**BMC Public Health (2024) 24:1107**

10.1186/s12889-024-18553-3

In the original publication of this article there was an incorrect author name. The incorrect and correct name are listed below. The publisher apologizes for the inconvenience caused to the authors.

Incorrect

A. K. Mohammed

Correct

Mohammed AK

