# Supplementary material for: Evaluating knowledge, attitude, and physical activity levels related to cardiovascular disease in Egyptian adults with and without cardiovascular disease: a community-based cross-sectional study
Source: BMC Public Health. 2024 Apr 22;24:1107. doi: 10.1186/s12889-024-18553-3 (PMC11036560; doi:10.1186/s12889-024-18553-3)
Supplement: Supplementary file 1 — Supplementary Material 1 [file 12889_2024_18553_MOESM1_ESM.docx]

**Supplementary**

| **Table S1. Factors affecting knowledge score among enrolled participants (N=463) & CVD patients (N=128) in this study** | | | | | | | |
| --- | --- | --- | --- | --- | --- | --- | --- |
| **CVD Knowledge Score** | | **NON-CVD** | | | **CVD** | | |
|  |  | **N** | **Mean (SD)** | **P-value** | **N** | **Mean (SD)** | **P-value** |
| **Gender** | Male | 208 | 21.38(7.4) | 0.602 | 98 | 19.65(7.4) | 0.206 |
|  | Female | 255 | 21.36(6.7) |  | 30 | 21.27(4.7) |  |
| **Age** | <45 | 11 | 21.36(6.6) | 0.645 | 23 | 21.7(3.8) | 0.316 |
|  | 45-65 | 2 | 19(2.8) |  | 4 | 21.75(3.2) |  |
|  | >65 | 450 | 21.4(7) |  | 101 | 19.58(7.5) |  |
| **Marital Status** | Single | 343 | 21.3(7.3) | 0.926 | 10 | 19.3(6.1) | 0.482 |
|  | Married | 117 | 21.5(6.3) |  | 113 | 19.9(7) |  |
|  | Divorced | 3 | 23(5.29) |  | 1 |  |  |
|  | Widowed | 0 |  |  | 4 | 23.5(2.4) |  |
| **Education** | No Formal Education | 0 |  | 0.361 | 9 | 21.67(4.3) | 0.701 |
|  | Primary Level | 0 |  |  | 3 | 21(3.5) |  |
|  | Preparatory Level | 0 |  |  | 5 | 23(3.4) |  |
|  | Secondary Level | 40 | 18.4(8.7) |  | 6 | 22.5(2.9) |  |
|  | University Level | 389 | 21.6(6.8) |  | 104 | 19.56(7.4) |  |
|  | Postgraduate | 34 | 22.7(5.9) |  | 1 |  |  |
| **Occupation** | Employed | 218 | 21.9(6.7) | 0.097 | 106 | 19.7(7.4) | 0.288 |
|  | Unemployed | 245 | 20.9(7.2) |  | 22 | 21.5(3.4) |  |
| **Social and Economic Status** | Low | 67 | 21.5(6.6) | 0.12 | 112 | 19.9(7) | 0.574 |
|  | Moderate | 372 | 21.2(7.1) |  | 15 | 20.5(5.9) |  |
|  | High | 24 | 23.8(6.5) |  | 1 |  |  |
| **Smoking Status** | Smoker | 423 | 21.4(7) | 0.499 | 81 | 20.3(6.5) | 0.389 |
|  | Non-smoker | 40 | 21.2(5.98) |  | 47 | 19.6(7.6) |  |

| ***Table S2. Factors affecting attitude score among enrolled participants (N=463) & CVD patients (N=128) in this study*** | | | | | | | |
| --- | --- | --- | --- | --- | --- | --- | --- |
| **CVD attitude Score** | | **NON-CVD** | | | **CVD** | | |
|  |  | **N** | **Mean (SD)** | **P-value** | **N** | **Mean (SD)** | **P-value** |
| Gender | Male | 208 | 67.2(9.3) | 0.895 | 98 | 63.4(7.4) | 0.073 |
|  | Female | 255 | 67.45(8.3) |  | 30 | 60.6(6.9) |  |
| Age | <45 | 11 | 68.3(5.97) | 0.479 | 23 | 63(7.2) | 0.785 |
|  | 45-65 | 2 | 63(2.9) |  | 4 | 65(5.5) |  |
|  | >65 | 450 | 67.3(8.8) |  | 101 | 62.6(7.5) |  |
| Marital Status | Single | 343 | 66.8(8.98) | 0.081 | 10 | 62.9(8.4) | 0.444 |
|  | Married | 117 | 68.9(7.8) |  | 113 | 62.82(7.4) |  |
|  | Divorced | 3 | 73(10.6) |  | 1 |  |  |
|  | Widowed | 0 |  |  | 4 | 59.3(3.86) |  |
| Education | No Formal Education | 0 |  | 0.954 | 9 | 60.2(6) | 0.42 |
|  | Primary Level | 0 |  |  | 3 | 64(2) |  |
|  | Preparatory Level | 0 |  |  | 5 | 60.4(7) |  |
|  | Secondary Level | 40 | 66.6(10.6) |  | 6 | 64.2(6.2) |  |
|  | University Level | 389 | 67.3(8.6) |  | 104 | 63.1(7.5) |  |
|  | Postgraduate | 34 | 68.6(7.9) |  | 1 |  |  |
| Occupation | Employed | 218 | 67.8(8.3) | 0.532 | 106 | 62.9(7.6) | 0.515 |
|  | Unemployed | 245 | 66.9(9) |  | 22 | 62.1(6.3) |  |
| Social and Economic Status | Low | 67 | 67(8.8) | 0.456 | 112 | 62.4(7.1) | 0.084 |
|  | Moderate | 372 | 67.5(8.8) |  | 15 | 64.8(8.4) |  |
|  | High | 24 | 65.6(8.3) |  | 1 |  |  |
| Smoking Status | Smoker | 423 | 67.57(8.8) | 0.031 | 81 | 63.3(7.7) | 0.281 |
|  | Non-smoker | 40 | 65(8.4) |  | 47 | 61.8(6.7) |  |
